# Effect of topdressing time on spring maize yield and nitrogen utilization in black soil of northeast China

**DOI:** 10.1038/s41598-023-38724-3

**Published:** 2023-07-22

**Authors:** Yu Zheng, Jinghong Ji, Shuangquan Liu

**Affiliations:** 1 Heilongjiang Academy of Agricultural Sciences Postdoctoral Programme, Harbin, 150086 China; 2 Heilongjiang Academy of Black Soil Conservation and Utilization, No. 368, Xuefu Road, Harbin, 150086 Heilongjiang People’s Republic of China

**Keywords:** Ecology, Environmental sciences

## Abstract

Topdressing time is crucial to achieving a high yield. To determine the optimum topdressing time for spring maize in the black soil of northeast China in the “one base and one topdressing” mode, the effects of topdressing time of nitrogen (N) fertilizer on maize yield, N utilization, and inorganic N residue and distribution were investigated by using ^15^N labeling technique. Four treatments were designed: no N fertilizer (N0), N fertilizer topdressing at jointing stage (N1), N fertilizer topdressing at belling stage (N2), and N fertilizer topdressing at tasseling stage (N3). The results showed that compared with N1 and N3, the maize yield, N uptake and N use efficiency (NUE) in N2 treatment significantly increased by 12.1% and 24.7%, 10.0% and 16.0%, and 26.4% and 38.9%, respectively (*P* < 0.05). The later the topdressing time, the more inorganic N remained in the soil profile (0–60 cm). The rate of potential N loss was higher when the topdressing time was too early or too late. Compared with N1, the residual amount of ^15^N in the soil profile (0–60 cm) of N2 and N3 treatments increased by 17.2% and 44.8%, respectively. The soil inorganic N (SIN) accumulation in the deep soil profile (40–60 cm) of N2 treatment decreased by 7.6% and 42.7%, respectively, as compared with N1 and N3. Therefore, the application of N fertilizer at the belling stage was beneficial to the high yield and efficient production of maize in the black soil region of Northeast China.

## Introduction

Maize is the largest grain crop in China, with a planting area of 4.13 × 10^7^ ha^−1^ and a yield of 2.61 × 10^8^ t in 2020^1,2^. The northeast region produces the most grain, accounting for 12.7% of China’s total maize production^3^. Maize is a high-demand N plant, and N is an important factor limiting its growth and yield^4^. In order to increase maize yield, excessive N application has become increasingly common, resulting in low N fertilizer utilization, soil quality decline, and high input production costs^5–8^. Precision fertilization based on crop fertilizer requirements, soil fertility capacity, and fertilizer performance, is essential to achieving high yield, improving fertilizer utilization efficiency, and promoting sustainable agriculture^9–11^.

Regarding to the N requirements of maize, the timing of N application significantly affects the physiological characteristics of maize^12–14^, split N application is conducive to N absorption during the grain filling stage, reducing N loss, maintaining soil N balance^15–17^. Previous studies noted that the method of immediate excessive N application or base application with topdressing during the seedling stage and later stages, which leads to problems such as excessive growth in the early stages, increased risk of lodging^18,19^, serious N deficiency disorder in later stages, early plant ageing, a shortened grain filling period, and a decreased in grain filling rate^20^. It is difficult to work with maize in the field manually or mechanically after the tasseling stage. Therefore, maize topdressing is challenging in agriculture practice. There have been many reports on the effects of N fertilizer optimization on maize yield and N utilization, but the reported application period of N fertilizer varies. Ding et al.^21^ argue that when the amount of fertilizer is not too high, N application during the jointing stage can promote the early form building of maize. Jiang et al.^22^ found that topdressing during the late milk stage could improve the number of ears and the thousand-grain weight, and increase the economic coefficient. Wang et al.^23^ reported that delaying N application until the tasseling stage could improve grain weight and crude protein content.

At present, the most common fertilization mode in northeastern China is 'one base and one topdressing' fertilization mode^24–26^, that is a portion of N fertilizer is applied at sowing as a base fertilizer, and another portion is applied as topdressing during the growing period. Studying the appropriate time for N fertilizer application in the "one base and one topdressing" fertilization mode has become an urgent issue in maize production in this region, it is necessary to further clarify the optimal stage for N fertilization. In addition, the use of ^15^N tracing technology to clarify the absorption, accumulation, distribution, and soil residue of ^15^N in plants and soil at different stages of N fertilization has not been reported in maize production in cold regions. Therefore, based on the ^15^N-labeled urea, a micro-plot field experiment was conducted to investigate the effects of N application at three key growth stages—the jointing stage, the belling stage, and the topdressing stage—on dry matter accumulation, N uptake and accumulation in plants, N fertilizer use efficiency, and soil residue of spring maize. The aim of the study was to find the best approach to N management and provide an empirical and theoretical basis for sustainable and high-yielding maize production.

## Materials and methods

### Experimental site

The experiment was carried out from 2020 to 2021. The experimental site was located at Binxian County Agricultural Technology Extension Center (45°46′N, 127°30′E), which belongs to the cold-temperate continental climate zone. During 1980s to 2021, the annual average temperature is 1.5 °C, the precipitation is 550 mm, the frost-free period is approximately 145 d, the sunshine duration is 2571.1 h, and the annual evaporation is 1586.8 mm. The soil type is medium fertility black soil, formed from loess deposition. The soil characteristics of the top 20 cm were pH 6.45, bulk density 1.31 g cm^−3^, organic C 30.6 g kg^−1^, alkaline hydrolysis N 141.6 mg kg^−1^, available phosphorus (Olsen-P) 38.3 mg kg^−1^and available potassium 169.2 mg kg^−1^.

### Experimental design

A randomized complete block design was adopted for this experiment. The variety of maize used was Zhengdan 958, and the planting density was 60,000 plants per hectare. “One base and one topdressing” mode is currently the most common form of fertilization for maize spring in Northeast China^24–26^, with 40% of nitrogen fertilizer is applied as a base fertilizer, and 60% is applied as top dressing during the growing period. In a previous study, we had determined that, the recommended optimal N level was 220 kg ha^−1^ ^27^. According to the agriculture production practice of local farmer and our research group, and weaken the influence of the experimental error caused by the microplot, the amount of nitrogen fertilizer need increased by 30% accordingly, the N fertilizer applied was set as 280 kg ha^−1^ to easy operation, with a base application of 112 kg ha^−1^ and a topdressing application of 168 kg ha^−1^.

Four treatments were set: (i) no N fertilizer application (N0), (ii) N topdressing at the jointing stage (N1), (iii) N topdressing at the belling stage (N2), and (iv) N topdressing at the tasseling stage (N3). Each treatment was repeated three times (Table 1).

**Table 1:** Nitrogen application stages and ratios of different treatments.

Treatment		Basal N (%)	Jointing stage (%)	Belling stage (%)	Tasseling stage (%)
N0		0	0	0	0
N1	I	40*	60	0	0
	II	40	60*	0	0
N2	I	40*	0	60	0
	II	40	0	60*	0
N3	I	40*	0	0	60
	II	40	0	0	60*

“*”Indicated ^15^N-labelled urea; “I” indicated ^15^N-labelled urea applied as basal fertilizer; “ II” indicated ^15^N-labelled urea applied as topdressing fertilizer. The same in Table 2.

Experimental trials were conducted in a test area measuring 39 m^2^ (0.65 m width, 10 m length, with 6 ridges), utilizing plot-scale experiments within the area on a micro-scale of 0.150 m^2^ (30 cm × 50 cm). A square metal bucket made of white iron was buried 50 cm underground and marked with a single plant to facilitate the trial. Except for the N0 treatment, the amount of N, phosphorus, and potassium fertilizer for each treatment was the same, with a dosage of N 280 kg ha^−1^, P_2_O_5_ 90 kg ha^−1^, and K_2_O kg ha^−1^. The ^15^N urea used was produced by the Shanghai Institute of Chemical Technology, with an abundance of 10.22% and an N content of 46%. The ordinary N fertilizer used was urea (N content 46%), the phosphorus fertilizer was calcium superphosphate (P_2_O_5_ content 46%), and the potassium fertilizer was potash (K_2_O content 60%).

### Soil and plant sampling

Soil samples were collected before sowing and after harvest. Before planting in Spring, soil cultivation layer (0–20 cm) samples were taken to test physical and chemical properties. After harvest in Autumn, soil samples (0–20 cm, 20–40 cm, 40–60 cm) were taken to be air-dried in the laboratory and crushed through a 100-mesh screen to determine the residue of N and ^15^N in soil under different treatments.

Plant samples were collected in both large and micro areas. Plant samples were collected a total of 5 times, before fertilizer application. Destructive sampling was used in micro areas. Three labeled plants were taken for each treatment (1 plant was taken for each replicate). The sampling dates were June 14th and 16th, June 28th and 29th, July 19th and 21st, August 23rd and 26th, September 26th and 29th in 2020 and 2021, respectively. These dates correspond to the seedling stage, the jointing stage, the belling stage, the tasseling stage and the maturity stage, respectively. The maize plant samplings were categorized by organ (such as stalk, leaf, grain, cob, etc.). All plant samples were washed with water and deionized water several times. After sterilizing at 105 °C for 30 min, the samples were dried at 75 °C to ensure consistent weight and weighed.

### Data analysis

The residual amount of N in soil and plant uptake were calculated by measuring the N content and ^15^N abundance in soil and plants. This information was then used to calculate the N utilization rate, residual rate and loss rate of the N fertilizer. The main parameters were calculated as follows^28,29^:

1 $$^{{{15}}} {\text{N}}\;{\text{atom}}\;\% \;{\text{excess }}\left( \% \right) =^{{{15}}} {\text{N}}\;{\text{abundance}}\;{\text{of}}\;{\text{sample}}/^{{{15}}} {\text{N}}\;{\text{labeled}}\;{\text{fertilizer}} -^{{{15}}} {\text{N}}\;{\text{natural}}\;{\text{abundance}}$$

(Note: the value of natural ^15^N abundance is 0.3663% in equation).

The percentage of N derived from fertilizer N (Ndff, %) was quantified:

2 $$\begin{aligned} {\text{Ndff }}\left( \% \right) & = {\text{atom}}\%^{{{15}}} {\text{N}}\;{\text{excess}}\;{\text{in}}\;{\text{plant}}\;{\text{or}}\;{\text{soil}}/{\text{atom}}\%^{{{15}}} {\text{N}}\;{\text{excess}}\;{\text{in}}\;{\text{fertilizer }} \\ & = {{\left( {^{{{15}}} {\text{N abundance}}\% {\text{ in plant }}{-}{\text{ natural}}^{{{15}}} {\text{N abundance}}} \right)} \mathord{\left/ {\vphantom {{\left( {^{{{15}}} {\text{N abundance}}\% {\text{ in plant }}{-}{\text{ natural}}^{{{15}}} {\text{N abundance}}} \right)} {\left( {^{{{15}}} {\text{N abundance in fertilizer}}\% \, {-}{\text{ natural}}^{{{15}}} {\text{N abundance}}} \right)}}} \right. \kern-0pt} {\left( {^{{{15}}} {\text{N abundance in fertilizer}}\% \, {-}{\text{ natural}}^{{{15}}} {\text{N abundance}}} \right)}} \times {1}00 \\ \end{aligned}$$

3 $$\begin{aligned} {\text{Plant}}\;{\text{total}}\;{\text{ N}}\left( {{\text{kg ha}}^{{ - {1}}} } \right) & = {\text{plant}}\;{\text{dry}}\;{\text{matter}}\left( {{\text{kg}}\;{\text{ha}}^{{ - {1}}} } \right) \times {\text{plant}}\;{\text{N}}\;{\text{concentration }}\left( {{\text{g}}\;{\text{kg}}^{{ - {1}}} } \right) \\ & \;\;\;\; + {\text{ grain}}\;{\text{dry}}\;{\text{matter}}\left( {{\text{kg}}\;{\text{ha}}^{{ - {1}}} } \right) \times {\text{grain}}\;{\text{N}}\;{\text{concentration}}\left( {{\text{g}}\;{\text{kg}}^{{ - {1}}} } \right)/{1}000 \\ \end{aligned}$$

4 $$\begin{aligned} {\text{Soil}}\;{\text{residual}}\;{\text{amount}}\;{\text{of}}^{{{15}}} {\text{N}}\left( {{\text{kg}}\;{\text{ha}}^{{ - {1}}} } \right) & = {\text{Ndff}}\;{\text{soil}}\left( \% \right) \times {\text{soil}}\;{\text{total}}\;{\text{N}}\left( \% \right) \\ & \;\;\;\; \times {\text{soil}}\;{\text{thickness}}\left( {{\text{cm}}} \right) \times {\text{soil}}\;{\text{bulk}}\;{\text{density}}\left( {{\text{g}}\;{\text{cm}}^{{ - {3}}} } \right) \times {1}00 \\ \end{aligned}$$

5 $$^{{{15}}} {\text{N}}\;{\text{recovery}}\;{\text{efficiency }}\left( {^{{{15}}} {\text{NRE}},\% } \right) =^{{{15}}} {\text{N}}\;{\text{in}}\;{\text{plant}}/^{{{15}}} {\text{N}}\;{\text{fertilizer}}\;{\text{application}}\;{\text{rate}} \times {1}00$$

6 $${\text{Apparent N recovery efficiency }}\left( {{\text{ANRE}},\% } \right) = \left( {{\text{N}}\;{\text{uptake}}\;{\text{in}}\;{\text{fertilized}}\;{\text{plot}}{-}{\text{N}}\;{\text{uptake}}\;{\text{in}}\;{\text{unfertilized}}\;{\text{plot}}} \right)/{\text{N}}\;{\text{application}}\;{\text{rate}}$$

7 $${\text{Potential}}\;{\text{N}}\;{\text{losses}}\left( \% \right) = {1}00{-}{\text{N}}\;{\text{recovery}}\;{\text{efficiency}}\left( \% \right){-}{\text{N}}\;{\text{residual}}\;{\text{efficiency}}$$

The software SPSS 21.0 (IBM, Armonk, NY, USA) was used to examine significant differences in all the indexes of the different treatments by one-way analysis of variance (ANOVA). All figures were generated using Sigmaplot 12.0 (Systat Software Inc., San Jose, CA, USA).

## Results

### Maize yield and biomass

The grain yield and biomass of maize were significantly affected by the application of N fertilizer and different topdressing time, which showed the same trend during two years (Table 2, *P* < 0.05). Compared with the CK treatment, the grain yield and biomass of maize under nitrogen fertilizer treatments significantly increased by 39.2–74.7%, and 39.0–66.5%. For different topdressing time, the grain yield and biomass of maize in N2 treatment significantly increased by 12.1% and 24.7%, and 10.2% and 19.2%, respectively, as compared with the N1 and N3 treatments.

**Table 2:** Effects of topdressing time on maize yield, biomass, N uptake and utilization.

Year	Treatment		Grain yield (t ha^−1^)	Biomass (t ha^−1^)	N uptake (kg ha^−1^)	^15^NRE (%)	Ndff (%)	Ndfs (%)	N residual (%)	N potencial loss (%)	
2020	N0		8.97e	17.18d	166.7d	–	–	–	–	–	–
	N1	I	14.35bc	26.89b	261.3abc	30.5b	24.8bc	75.2 cd	42.8ab	26.7ab	28.5a
		II	13.92bc	25.06bc	270.2abc		25.4b	74.6d	39.2b	30.3a	
	N2	I	16.12a	29.24a	284.5ab	38.1a	22.5 cd	77.5bc	44.1ab	17.8abc	14.9b
		II	15.34ab	27.12ab	292.7a		31.1a	68.9e	50.0ab	11.9bc	
	N3	I	12.43d	24.2c	252.1bc	28.1b	20.4de	79.6ab	51.3ab	20.6abc	15.3b
		II	12.92 cd	24.32c	246.0c		18.3e	81.7a	61.9a	10.0c	
2021	N0		7.87e	14.80c	137.7d	–	–	–	–	–	–
	N1	I	12.57abc	23.57a	225.5bc	29.4b	23.2a	76.8b	38.4ab	32.2ab	34.7a
		II	11.53bcd	21.55b	230.1bc		26.2a	73.8b	33.4b	37.2a	
	N2	I	13.12ab	24.03a	241.8b	37.6a	23.0a	77.0b	42.3ab	20.1ab	15.4b
		II	14.08a	24.61a	267.4a		27.1a	72.9b	51.8ab	10.6b	
	N3	I	11.15 cd	21.10b	228.7bc	26.3b	16.8b	3.2a	49.8ab	23.9ab	19.8b
		II	10.52d	20.13b	209.9c		18.2b	81.8a	58.1a	15.6ab	
Aver. 2020–2021	N0		8.42d	15.99c	152.2e	–	–	–	–	–	–
	N1	I	13.46ab	25.23a	243.4 cd	29.9b	24.0b	76.0b	40.6 cd	29.5a	31.7a
		II	12.72bc	23.30b	250.2bc		25.8ab	74.2bc	36.3d	33.8a	
	N2	I	14.62a	26.63a	263.2ab	37.8a	22.8b	77.2b	43.2c	19.0b	15.2b
		II	14.71a	25.86a	280.4a		29.1a	70.9c	50.9b	11.3c	
	N3	I	11.79c	22.65b	240.4 cd	27.2b	18.6c	81.4a	50.6b	22.2b	17.5b
		II	11.72c	22.22b	227.5d		18.2c	81.8a	60.0a	12.8c	

Different lowercase letters indicate the significant difference among different fertilization treatments according to LSD test (*P* < 0.05).

### Nitrogen uptake of maize in different growth stages

**Figure 1:**
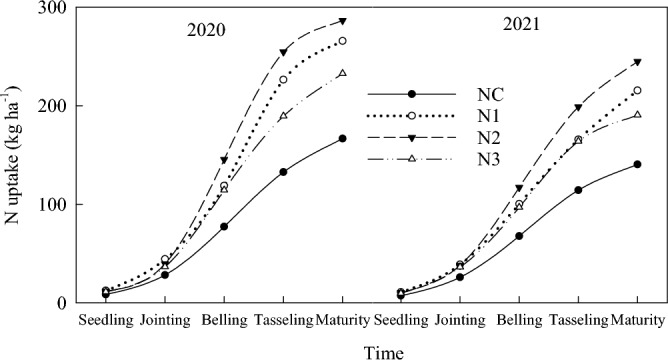
N uptake of maize at different growth stages under different treatments.

There were two peaks in N uptake over the two years. One is from the joint stage to the belling stage, and the other is from the belling stage to the grain filling stage (Fig. 1). The curve of the second peak period rises particularly sharply. In addition, the vegetative growth and reproductive growth of maize correlate, and N demand is urgent. From the seedling stage to the maturity stage, N uptake in N0, N1, N2 and N3 treatment was ranged from 7.8 to 152.2, 11.5 to 246.8, 11.1 to 271.6, and 10.4 to 234.2 kg ha^−1^, respectively (Fig. 1). The percentages of N uptake in the seedling stage, the seedling stage to jointing stage, the jointing stage to belling stage, the belling stage to tasseling stage and the tasseling stage to maturity stage were 4.6%, 11.6%, 30.2%, 34.9% and 18.7%, respectively.

**Figure 2:**
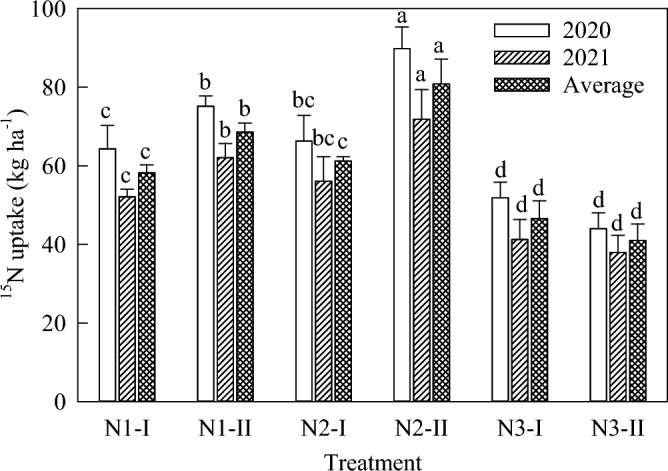
^15^N uptake of maize under different treatments at the maturity stage.

The N uptake of the maize was significantly affected by the topdressing time (Fig. 2, *P* < 0.05). The N2 treatment, in which N fertilizer was topdressed at the belling stage (V12), had the most beneficial effect. The N uptake in the mature stage increased by 10.0% and 16.0% as compared with that in the jointing stage and tasseling stage, respectively (*P* < 0.05). The ^15^N uptake and fertilizer N uptake ratio of maize was significantly affected by different topdressing times (Fig. 2). The ^15^N uptake of N1, N2 and N3 treatments at mature stage was 63.4, 71.0 and 43.8 kg ha^−1^, which accounted for 25.7%, 26.1% and 18.7% of the total N uptake of maize at the mature stage, respectively. There are 18.2–29.1% of the N uptake by maize came from the application of N fertilizer, and 70.9–81.8% came from the soil (Table 2).

### Nitrogen use efficiency and nitrogen fate of maize

**Figure 3:**
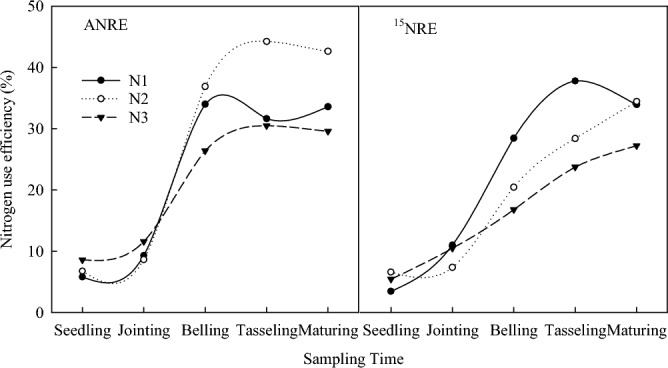
Dynamic changes of N use efficiency at different growth stages of maize.

**Figure 4:**
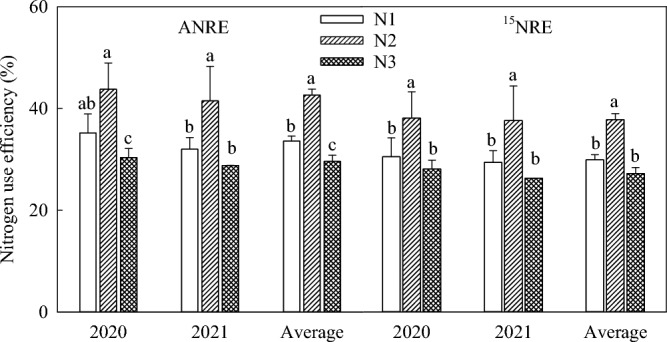
Apparent N use efficiency and ^15^N recovery of different treatment.

Nitrogen use efficiency was significantly affected by different nitrogen fertilizer treatments and different topdressing time (Fig. 3). The NUE was the lowest in the seedling stage, and increased slowly from the seedling stage to jointing stage, rapidly from the jointing stage to belling stage, and fluctuated from the belling stage to maturity stage. From the seedling stage to maturity stage, N apparent availability ranged from 5.8 to 44.3%. The utilization rate of ^15^N was between 3.5 and 38.3%; N fertilizer apparent N utilization rate (ANRE) was slightly higher than ^15^N recovery rate (NRE), but the trend was consistent. The N2 treatment significantly increased N apparent use efficiency, which was 27.1% and 44.3% higher than that in N1and N3 treatments, respectively (Fig. 4, *P* < 0.05). According to the ^15^N recovery rate (Fig. 4), the average value of N2 treatment was 26.4% and 38.9% higher than in N1 and N3 treatments, respectively. The soil residual rate of ^15^N was 38.5% in N1 treatment, 47.1% in N2 treatment and 55.3% in N3 treatment; the average potential loss rate of ^15^N was 31.7% in N1, 15.2% in N2, and 17.5% in N3 (Table 2). There was no significant difference between N2 and N3, but there were significant differences between N2 and N3 with N1 cross the three categories.

### Nitrogen distribution in maize organs

**Figure 5:**
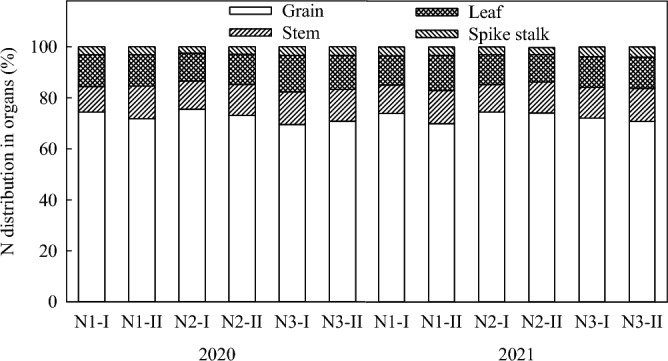
N distribution in maize organs under different treatments.

**Figure 6:**
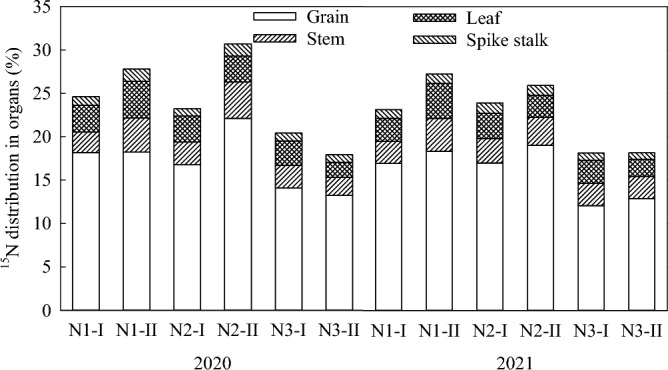
^15^N distribution in maize organs under different treatments.

Nitrogen was mainly distributed in the grains, followed by leaves, stems and cob, with an average distribution of 72.7%, 12.4%, 11.3% and 3.6%, respectively (Fig. 5). There was little difference in the proportion of N between stem and leaf. In the nitrogen of the above organs, ^15^N accounted for 13.1–20.6%, 2.3–3.8%, 1.9–4.1%, and 0.8–1.3% of total nitrogen content, respectively (Fig. 6). In N1 treatment, the distribution ratio of ^15^N in grain, leaf, stem and cob was 17.9%, 3.2%, 3.5% and 1.1%. In N2 treatment, the distribution ratio of ^15^N was 18.1%, 3.2%, 2.9% and 1.1%. In N3 treatment, the distribution ratio was 13.1%, 2.4%, 2.3% and 0.9%. In conclusion, N1 and N2 treatments promoted N transfer to grains, while N3 treatment reduced N accumulation in grains, thus reducing N availability.

### Accumulation and distribution of ^15^N in soil profile

**Figure 7:**
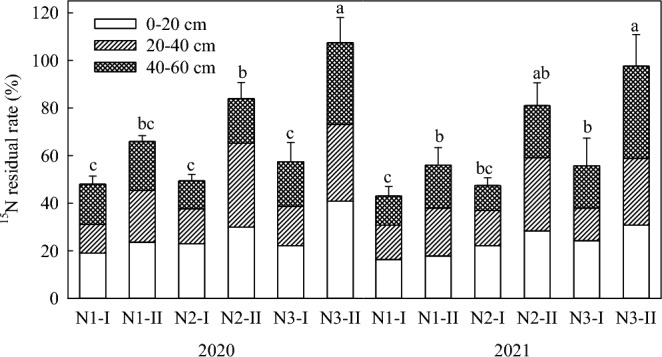
Accumulation and distribution of ^15^N in soil profiles under different treatments.

The residual N in the soil profile was affected by the different application of N fertilizer (Fig. 7). The residual N decreased with soil layer. However, when the N3 topdressing period was delayed, the uptake and utilization rate of maize decreased, and the residual N in the soil layer increased accordingly. The residual amount of ^15^N in the soil profile (0–60 cm) of N1, N2 and N3 treatments were 56.9, 66.7 and 82.4 kg ha^−1^, respectively. Compared with N1 treatment, N2 and N3 increased by 17.2% and 44.8%, respectively, while N1 and N2 had no significant difference. N3 was significantly different from N1 and N2 (*P* < 0.05). In terms of soil depth, the residual amount of ^15^N in 0–20 cm, 20–40 cm and 40–60 cm treated with N1 was 28.3, 19.0 and 9.6 kg ha^−1^, respectively. In N2 treatment 0–20 cm, 20–40 cm and 40–60 cm, the residual amount of ^15^N was 38.1, 19.7 and 8.9 kg ha^−1^, respectively. In 0–20 cm, 20–40 cm and 40–60 cm treated with N3, the residual amount of ^15^N was 43.4, 23.6 and 15.4 kg ha^−1^, respectively. In the topsoil layer of 0-20 cm, the residual ^15^N amount increased by 53.4% and 13.9% in N3 compared to N1 and N2, respectively. In the 20–40 cm layer, the residual ^15^N amount increased by 24.2% and 19.8% in N3 compared to N1 and N2, respectively. In the 40–60 cm soil layer, the residual ^15^N amount decreased by 7.3% and 42.2% in N2 compared to N1 and N3, respectively. It is evident that the N2 treatment resulted in a decrease in nitrogen residue in the 40–60 cm soil layer, while N3 treatment led to an increase in nitrogen residue in the same layer. The N1 treatment was intermediate between N2 and N3.

## Discussion

### Effect of topdressing time of nitrogen fertilizer on maize yield

Yang et al.^34^ found that under the conditions of applying 225 kg ha^−1^ of nitrogen fertilizer, nitrogen application in the early stages was conducive to increasing the nutrient reservoir. In contrast, application in the later stages was beneficial to the accumulation of dry matter in the later stages and promoted the transfer of dry matter from the nutrient organ to the grains. However, they also reported that if fertilization is applied too early (such as with only basal or seedling fertilizer) or too late (such as in the filling stage), it harms yield. Amanullah et al.^32^ reported that the timing of nitrogen fertilizer application significantly affects nitrogen accumulation in crops, thereby affecting crop growth, development, and grain yield. Shang et al.^33^ found that the accumulation of nitrogen in winter wheat plants under different nitrogen application periods demonstrated a high utilization rate of straw in the early stages of application. In contrast, the utilization rate of grains was higher in the later stages of application.

The results of this experiment are consistent with the study conducted by Yang et al.^34^. Under unchanged total nitrogen amount, in the ecotone of black soil in cold areas, the application of nitrogen fertilizer during the tasseling stage (N2, V12) had the highest yield and biomass of maize. The increase in yield was 12.1% and 24.7% compared to applying nitrogen fertilizer during the jointing stage (N1, V6) and the tasseling stage, respectively. Biomass increased by 10.2% and 19.2%, respectively (Table 2). Applying the base fertilizer and the jointing stage topdressing fertilizer can cause a shortage of fertilizer in the later stage of crop growth, affecting the accumulation and distribution of dry matter after flowering. Topdressing during the flowering stage led to a lower proportion of nitrogen fertilizer in the early stage and a higher proportion in the later stage, suppressing of assimilate accumulation during plant nutrition growth, leading to insufficient assimilates for reproductive growth in the later stage. Increasing nitrogen application in the tasseling stage reduce the distribution rate of ^15^N in leaves and stems, and increase its distribution rate in grains, which is conducive to improving grain yield.

### Effects of topdressing time on N uptake and N use efficiency of maize

The proportion of nitrogen accumulation during different growth stages of maize is as follows: 26.3% from emergence to jointing, 35.6% from jointing to tasseling, 13.0% from tasseling to silking, 18.0% from silking to grain filling, and 7.1% from grain filling to maturity^35^. According to a study by Qi et al.^36^, nitrogen distribution in maize varies among organs: 58–68% in grains, 22–33% in leaves, 4–8% in cobs, and 3%-8% in stems. Notably, the nitrogen absorption in grains is significantly higher than that in other organs, and the application of nitrogen fertilizer increases the nitrogen absorption in all organs.

The present study shows that the percentage of nitrogen absorption during different growth stages of spring maize from emergence to jointing, jointing to tasseling, tasseling to silking, silking to grain filling, and grain filling to maturity is 4.6%, 11.6%, 30.2%, 34.9%, and 18.7%, respectively. This result is lower than that obtained by Wang et al.^37^, who reported that the nutrient absorption of summer maize during the emergence to jointing stage was 26.3%, primarily due to the low temperature and high probability of drought in the early Spring of the black soil area of Northeast China, resulting in slow nutrient release and low nutrient absorption during the emergence to jointing stage. At maturity, nitrogen was mostly concentrated in the grains, followed by the leaves, the stems and the cobs, with little difference between stems and leaves. In the N1 and N2 treatments, the contribution rates of ^15^N in the grains were 17.9% and 18.1%, respectively. In the N3 treatment, it was 13.1% (Fig. 6), indicating that applying nitrogen fertilizer during the jointing or the tasseling stage increases the nitrogen accumulation in maize grains during the maturity stage and its contribution rate. However, if the amount of nitrogen applied is insufficient during the early growth stage, the subsequent application of nitrogen fertilizer during the silking stage may negatively affect the formation and yield in the later stage, and leading to wasted fertilizer due to reduced nitrogen absorption in the later stage.

The optimization of nitrogen fertilizer management significantly affects the absorption and utilization of nitrogen fertilizer and soil nitrogen by crops, Wang et al.^37^ found that under late harvesting conditions, the nitrogen use efficiency increased by 3.5–6.9%, and the agronomic efficiency increased by 0.8 to 1.6 kg kg^−1^. Wu et al.^38^ demonstrated through ^15^N labeling experiments on summer maize that the recovery rate from one-time application of labeled nitrogen fertilizer ranged from 41.2 to 47.8%, with residual nitrogen rates of 40.7 to 47.5%. In this study, we used the ^15^N tracing technique to investigate the dynamic changes in nitrogen fertilizer utilization efficiency during different growth stages of maize. The results showed that the ^15^N recovery rate ranged from 27.2 to 38.1%, with a residual nitrogen rate of 38.5–55.3%. Compared with the results of Wu et al.^38^, the nitrogen fertilizer recovery rate was significantly lower, while the nitrogen fertilizer residual rate was higher. The analysis of the reasons can be attributed to several factors. Firstly, the experimental conditions involved field micro-area rain-fed agriculture, which naturally leads to variations in climate that can impact the absorption and utilization of nitrogen by maize. Additionally, the chosen soil type for this experiment was typical black soil, renowned for its rich nutrient content. Moreover, in order to compensate for disparities between micro-area and large-field experiments, the nitrogen application rate in this study was set at 280 kgN·hm^−2^, surpassing the maximum nitrogen fertilizer rate suggested by Wu^38^. Consequently, these combined factors contribute to the observed outcomes.As a result, this study exhibited a low ^15^N recovery rate and a high ^15^N residual rate. Alternatively, this outcome could indicate that the conventional nitrogen application rate in the region is excessively high, necessitating further research to determine the appropriate nitrogen application rate. Thus, it is imperative to optimize nitrogen management practices and refine recommendations in order to enhance the efficiency of nitrogen utilization in maize production. In this study, the nitrogen use efficiency of N2 treatment was 26.4% and 38.9% higher than that of N1 and N3 treatments, respectively. This may be attributed to the small biomass of maize in the early growth stage, which cannot absorb all the nitrogen fertilizer applied. After the emergence of tassels, the natural aging of leaves can decrease the photosynthetic capacity and nutrient absorption. Therefore, on the basis of sufficient nitrogen supply in the early growth stage, the matching of nitrogen fertilizer application with maize nitrogen requirements during the critical period of the earing stage can maximize nitrogen accumulation in maize.

### Effect of time of topdressing nitrogen fertilizer on residual and distribution of soil inorganic nitrogen

The accumulation of inorganic nitrogen in soil is related to soil nitrogen supply capacity, nitrogen fertilizer application rate and timing, and the ability of crops to absorb soil nitrogen^37^. In this experiment, ^15^N tracing technology was used to quantitatively study the residual effects of nitrogen fertilizer in soil after harvest under different nitrogen application timings with the same amount of nitrogen fertilizer applied at the jointing stage, tasseling stage and silking stage of spring maize. The results showed that the total residual amount of ^15^N in the 0–60 cm soil layer was ranged from 56.9 to 82.4 kg ha^−1^ for all treatments under different nitrogen application timings, and the residual amount of nitrogen fertilizer gradually decreased with increasing soil depth. As the nitrogen application timing was delayed, the residual amount of ^15^N in the 0–60 cm soil layer generally increased. Since the base fertilizer was applied to the soil when the maize was sown, the residual amounts of base fertilizer in different soil depths reflect the effect of different nitrogen application timings on the transport of nitrogen fertilizer. When only base fertilizer ^15^N urea was applied, the amount of ^15^N residual in the plow layer soil (0–20 cm) of N3 treatment increased by 53.4% and 13.9% compared with N1 and N2 treatments, respectively; in the 20–40 cm soil layer, the amount of ^15^N residual in N3 treatment increased by 24.2% and 19.80% compared with N1 and N2 treatments, respectively; in the 40–60 cm soil layer, the amount of ^15^N residual in N2 treatment decreased by 7.3% and 42.2% compared with N1 and N3 treatments, respectively.

The total residual amount of inorganic nitrogen was highest for N3, followed by N2 and N1 (Fig. 7), and the potential nitrogen loss rate was highest for N1, followed by N3 and N2 (Table 2). This indicates that the early nitrogen application timing of N1 treatment resulted in limited early growth and nitrogen absorption of maize, which increased the probability of nitrogen loss due to volatilization, leaching, nitrification, denitrification, etc., ultimately resulting in a lower residual amount of nitrogen. The late nitrogen application of N3 treatment increased the risk of nitrogen accumulation and leaching in the soil surface layer and downward. The nitrogen application timing of N2 treatment satisfied the dual needs of maize nutrition and reproductive growth, promoted spike differentiation, and promoted nitrogen absorption and utilization by maize. Moreover, it enabled a high accumulation of nitrogen in the later stage of growth, met the requirements of maize for material synthesis, improved nitrogen use efficiency, promoted dry matter accumulation, and ultimately significantly increased yield. This finding is similar to the results of Liu et al.^40^ and is basically consistent with Nelson et al. ^41^ and Skonieski et al. ^42^.

## Conclusions

The maize yield and nitrogen utilization was significantly affected by the topdressing time under the “one base and one topdressing” mode in the black soil maize ecological zone in the cold region of Northeast China. Among different topdressing time, the N fertilizer topdressing at the belling stage significantly increased maize yield, improved N uptake and utilization efficiency, reduced the accumulation and potential loss rate of inorganic N in the deep soil profile. Therefore, the application of N fertilizer at the belling stage is recommended as an effective measure for maize N fertilization in the region.

### Statement

Appropriate permission was taken for the collection of plant/seed, and all the guidelines were followed while the collection of the plant/seed.

## Data Availability

The original data can be obtained from the corresponding author upon reasonable request.
